# Development of Halloysite Nanotube-Infused Thermoset Soybean Bio-Resin for Advanced Medical Packaging

**DOI:** 10.3390/polym16121616

**Published:** 2024-06-07

**Authors:** Shahab Saedi, Abdus Sobhan, Magdalene Hoff, Siqun Wang, Kasiviswanathan Muthukumarappan

**Affiliations:** 1 Department of Agricultural and Biosystems Engineering, South Dakota State University, Brookings, SD 57007, USA; shahab.saedi@gmail.com (S.S.); abdus.sobhan@jacks.sdstate.edu (A.S.);; 2 College of Agriculture and Applied Sciences, Alcorn State University, Lorman, MS 39096, USA; 3 Center for Renewable Carbon, The University of Tennessee, Knoxville, TN 37996, USA; swang@utk.edu

**Keywords:** soybean oil, thermoset resin, halloysite nanotubes, medical packaging

## Abstract

The development of eco-friendly, mechanically stable, and biocompatible materials for medical packaging has gained significant attention in recent years. Halloysite nanotubes (HNTs) have emerged as a promising nanomaterial due to their unique tubular structure, high aspect ratio, and biocompatibility. We aim to develop a novel soybean oil-based thermoset bio-resin incorporating HNTs and to characterize its physical and functional properties for medical packaging. Soybean oil was epoxidized using an eco-friendly method and used as a precursor for preparing the thermoset resin (ESOR). Different amounts of HNTs (0.25, 0.50, and 1.0 wt.%) were used to prepare the ESOR/HNTs blends. Various characteristics such as transparency, tensile strength, thermal resistance, and water absorption were investigated. While incorporating HNTs improved the tensile strength and thermal properties of the ESOR, it noticeably reduced its transparency at the 1.0 wt.% level. Therefore, HNTs were modified using sodium hydroxide and (3-Aminopropyl) triethoxysilane (APTES) and ESOR/HNTs blends were made using 1.0 wt.% of modified HNTs. It was shown that modifying HNTs using NaOH improved the transparency and mechanical properties of prepared blends compared to those with the same amount of unmodified HNTs. However, modifying using (3-Aminopropyl) triethoxysilane (APTES) decreased the transparency but improved the water absorption of prepared resins. This study provides valuable insights into the design of HNT-based ESOR blends as a sustainable material for medical packaging, contributing to the advancement of eco-friendly packaging solutions in the healthcare industry.

## 1. Introduction

The function of medical packaging is of the utmost importance in preserving the effectiveness and integrity of healthcare supplies, medical devices, and pharmaceutical products [1]. In recent years, there has been a growing emphasis on sustainability within the healthcare sector, driven by environmental concerns [2]. Furthermore, there is a rising demand for safeguarding against physical damage and external contaminants, which can prevent the entry of moisture and oxygen while preserving packaged healthcare materials [3]. Epoxy resins are now finding new applications due to their outstanding performance and unique characteristics, such as high mechanical strength and chemical resistance [4]. Much research has been conducted on their eco-friendly nature and mechanical stability to confirm their commercial applications in the chemical and pharmaceutical sectors [4,5,6,7,8,9,10,11]. 

Soybean oil is an example of a vegetable oil that has gained popularity as a biobased feedstock due to its low price, lack of toxicity, and widespread availability [12]. It is composed of triglycerides, diglycerides, monoglycerides, and phosphoglycerides. Among them, triglycerides are the major components of vegetable oils (93–98%) [13,14]. Triglycerides are triesters in which glycerol joins with three fatty acids as a triol. Fatty acids attached to the structure of glycerol in naturally occurring vegetable oils usually have 14–22 carbon atoms and 0–3 unsaturated double bonds [15,16]. When an oxygen atom is added to a double bond, a three-membered ring called “epoxide” is formed and the process is known as epoxidation. Epoxidizing the double bonds present in soybean oil produces a substance known as epoxidized soybean oil (ESO). This material is extensively utilized as a bio-based plasticizer for PVC and PLA. Furthermore, the transformation of this raw material into epoxy resins has gained significant interest among researchers, as these resins have the potential to replace petroleum-based resins [5,12]. In general, epoxy resins derived from soybean oil provide a more environmentally friendly and economically viable alternative to conventional epoxy resins [17]. 

However, there are some problems with their use. These include a longer curing time, lower mechanical properties, less resistance to moisture, and a higher curing temperature. For instance, they may not be as robust as epoxy resins derived from petroleum [18]. The lower mechanical strength of vegetable oil-based epoxy resins could be due to the low cross-linking density and higher chain flexibility [8,18]. To extend the use of eco-friendly and sustainable thermoset resins derived from soybeans, their primary limitations, like low mechanical strength, must be overcome.

In this context, halloysite nanotubes (HNTs) could be an option worth considering. HNTs are mineral-based natural nanotubes with desired features such as a large surface area, mechanical strength, thermal stability, and low price. As a potential reinforcement for a wide variety of composite films, HNTs have been the subject of extensive research [19]. In a study [20], the rheological and adhesive properties and joint strength of epoxy oligomer–halloysite systems were investigated. They reported a 30% increase in joint strength by adding 2 wt.% of HNTs. It was mentioned that HNTs are effective in improving the adhesive properties of pressure-sensitive polyisobutylene (PIB)-based adhesives [21],. In a more relevant study to ours [22], a flexible, transparent resin with a low glass-transition temperature using hemp seed (*Cannabis sativa* L.) oil was prepared. They reported that at the optimum concentration of 0.50 wt.% in the HNTs’ filler, a simultaneous increase in stiffness, strength, ductility, and toughness was observed in comparison to the unfilled cured resin. However, HNTs are prone to aggregation because of their tubular shape and the presence of surface hydroxy groups, especially when combined with non-polar polymeric matrices. The mechanical properties of the polymer matrix are severely compromised by this aggregation, since the clustered HNT serves as sites of stress concentration, reducing the composite film’s overall strength. Furthermore, these aggregated HNTs significantly reduce the transparency of the resulting composite films. Additionally, the strong hydrophilicity of HNTs makes them a good absorber of water and moisture, further weakening the mechanical and barrier properties of the resulting blends. In this regard, different methods have been applied to modify the halloysite before mixing it with the polymer matrix. As reported in previous studies [23], alkali modification changes the zeta potential of HNTs from 0.18 ± 0.6 mV to −35.2 ± 2.8 mV, implying that alkali modification brings about a significant negative surface charge for HNTs. Halloysite is frequently modified with amino silane to improve its compatibility with hydrophobic polymer matrices. Amino silane modification involves functionalizing the surface of the halloysite with molecules of amino silane that contain both hydrophobic alkyl chains and polar amino groups. The hydrophobic properties of the alkyl chains are compatible with the hydrophobic polymer matrix, resulting in improved interfacial adhesion and mechanical properties of the nanocomposites [24,25,26,27]. Different treatment methods, including silane modification, were utilized to improve the dispersibility of HNTs in the epoxy resin matrix. It was found that the chemical treatment of HNTs helps their dispersion and results in enhanced mechanical properties of the halloysite–epoxy blend [28]. Silane modification was used to improve the dispersibility of HNTs in unsaturated polyester resins. It was shown that the modified HNTs appeared as uniformly dispersed halloysites, whereas the pristine HNTs formed skewed-like clusters in the unsaturated polyester nanocomposite [29]. Halloysite nanotubes (HNTs) were modified with 3-glycidyloxypropyltrimethoxysilane (GLYMO), 3-aminopropyltrimethoxysilane (APTES), and 2,2-Bis [4-(glycidyloxy) phenyl] propane (DGEBA) and incorporated in the epoxy resin matrix to enhance its mechanical properties. The results showed that the modification of HNTs enhanced their dispersion and the cross-linking in the epoxy resin matrix [30]. In our previous research, we demonstrated a successful sustainable pathway to epoxidized soybean oil using green solvent and raw materials and converting it to a thermoset resin [17,31]. To improve the mechanical, thermal, and water absorption properties of the aforementioned resins, HNTs were utilized, and the chemical modification approach was implemented to improve the dispersibility and compatibility of HNTs in the resin matrix. Epoxy resin was derived from soybean oil, and different amounts of HNTs were used to prepare soybean ESOR/HNT blends for future medical and healthcare applications. In the present work, we investigate the effect of the concentration and surface modification of halloysite on the properties of prepared ESOR. Our hypothesis for this research is that at higher concentrations of HNTs, the mechanical performance of the ESOR/HNT blend as well as its transparency decrease due to the aggregation of HNTs. The alkali modification of HNTs enhances their dispersibility in the ESOR and improves their transparency. Amino silane modification reduces the water absorption of HNTs and improves the water resistance of the resulting blend. Their transparency, visible light transmittance, mechanical strength, water absorption, and thermal strength are investigated. 

## 2. Materials and Methods

### 2.1. Materials

Refined soybean oil was supplied by Commercial and Trading Co., Ltd., located in Volga, SD, USA. Choline chloride (AR, 98.5%), formic acid (AR, 88%), sodium hydroxide (AR, 99%), acrylic acid, hydrogen peroxide (AR, 30%), oxalic acid dihydrate (AR, 99%), hydrochloric acid (AR, 99%), sodium hydroxide, and (3-Aminopropyl) triethoxysilane (APTES), and halloysite nanotube were all purchased from Sigma-Aldrich (St. Louis, MO, USA). 

### 2.2. Modification of Halloysite 

The alkaline modification of HNTs was performed according to the literature [23] with some modifications. To accomplish this, 20 g of NaOH (6 M) solution was prepared by dissolving 4.8 g of NaOH in 15.2 g of distilled water. Next, 2 g of HNTs was weighed, added to the solution, and sonicated using a probe-type sonicator (VCX 750, Sonics & Materials Inc., Newtown, CT, USA) at an amplitude of 60% (450 W; 20 kHz) for one hour. To avoid a rapid increase in temperature, the sonication treatment was carried out at 4 °C in a jacketed beaker. The modified HNT was designated as Al-HNT. Halloysite was modified using APTMS according to a technique reported in the literature [19]. Then, 200 mL of toluene and 10 g of halloysite (Hal) were mixed and sonicated for 20 min. The mixture was then heated and stirred until the temperature reached 60 °C. Once the mixture reached 60 °C, 10 mL of APTMS was added, and the reaction temperature was increased to 120 °C and maintained under a nitrogen atmosphere at 120 °C for another 24 h. The finished product was then cooled to room temperature, separated, and washed three times with clean toluene and ethanol. The obtained product was dried at 60 °C and designated as HNT@NH_2_.

### 2.3. Epoxidation of Soybean Oil

The preparation of the deep eutectic solvent and epoxidation of soybean oil were carried out according to a previously reported method [32]. To prepare the deep eutectic solvent (DES), 3.668 g of acetylcholine (AC) and 2.5692 g of oxalic acid (OA) were dissolved in 40 mL of deionized water and poured into a volumetric flask. Deionized water was added to the flask until the volume of the mixture reached 50 mL. The components were thoroughly mixed to ensure complete dissolution. For the epoxidation process, 20 g of soybean oil and 8.014 mL of DES were mixed in a beaker. Then, 11.36 g of formic acid was added slowly while the mixture was being stirred constantly. Next, 50 g of hydrogen peroxide (H_2_O_2_) was added dropwise over the next 15–20 min using a burette. The resulting mixture was stirred for 8 h at 35 °C. The mixture was then centrifuged for 10 min and washed with water three times. The supernatant was taken out, saved, and dried using MgSO_4_ as a drying agent to remove the remaining water. 

### 2.4. Thermoset Resin Preparation

**Figure 1:**
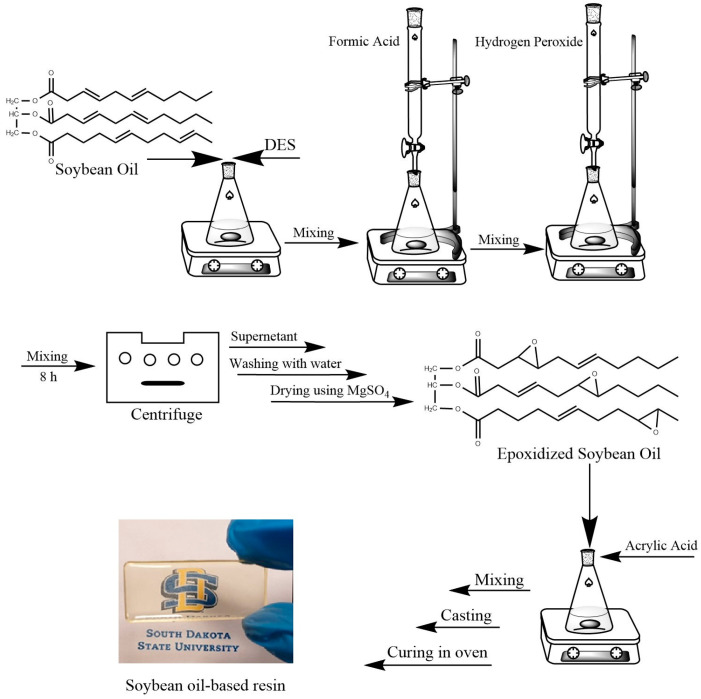
Schematic representation of the preparation procedure of soybean oil-based resins.

To prepare the ESOR, equivalent amounts of acrylic acid and epoxidized soybean oil were mixed in a beaker and stirred for five minutes. Then, 4 g of the mixture was poured into silicone molds (5 cm × 2 cm), and they were covered by a glass plate and placed gently in an oven preheated to 90 °C. The temperature ramping started by gradually raising the oven temperature, over two hours, from 90 °C to 100 °C. After another 2 h, the temperature increased to 110 °C; after an additional 2 h, it reached 120 °C. The temperature was maintained at 120 °C for the next 4 h to ensure that the resin was fully cured. The molds were then taken out of the oven and allowed to cool naturally. A high degree of cross-linking and the promotion of the necessary chemical processes were the goals of the temperature ramping procedure, which produced completely cured resins with the desired qualities. For ESOR/HNT blends, 0.25, 0.50, 0.75, and 1.0 wt.% of HNTs (based on the ESO) were added to the ESO and sonicated for 15 min. The acrylic acid was added, and the same procedure was repeated to obtain the cured thermoset resin. As described for the unmodified HNTs, 1.0 wt.% of alkali-treated and amino silane-modified HNTs was added, and the same procedure was followed. A schematic representation of the preparation procedure for thermoset resins is shown in Figure 1. The composition of the prepared blend resins is shown in Table 1.

### 2.5. UV–Vis Spectroscopy

The UV and visible light barrier properties of the ESOR/HNT blends were measured using a Vis spectrophotometer (Genesys 50 UV-Vis, Thermo Scientific, Madison, WI, USA) by measuring the light transmittance in the range of 350–700 nm. Samples were placed in the spectrophotometer cell. The air was used as the reference. 

### 2.6. FT-IR

A Fourier transform infrared (FT-IR) spectrometer (Spectrum Two, Perkin Elmer, Waltham, MA, USA) was employed to collect the FT-IR spectra of the HNTs and ESOR/HNT blends, and the spectra were recorded in the scan range of 4000–400 cm^−1^.

### 2.7. Optical Microscopy 

Micrograph images were acquired with a Carl Zeiss Axioskop 2 Mat Microscope, (Carl Zeiss Inc., Thornwood, NY, USA) fitted with a digital camera.

### 2.8. Mechanical Test

The mechanical strength of the obtained ESOR/HNT samples was evaluated using an Instron universal testing machine (Model 5565, Instron Engineering Corporation, Canton, MA, USA) under ASTM method D 882-88 [33]. The machine was operated with an initial grasp separation of 50 mm and a crosshead speed of 50 mm/min.

### 2.9. Thermal Stability 

Thermogravimetric analyses (TGA) were conducted using a Pyris 1 thermogravimetric analyzer (Perkin-Elmer, USA). The decomposition profile of the samples was investigated from 50 to 600 °C with a heating rate of 10 °C/min under 20 mL/min of nitrogen gas flow using about 10 mg of the sample. The decomposition temperature of each resin was determined by differentiating the thermographs acquired from the TGA into weight loss rates, referred to as differential TG (DTG) thermograms.

### 2.10. Water Absorption Tests

The procedure for conducting the water absorption tests was followed as outlined [34]. Prepared rectangular resins were placed in 200 mL beakers, each filled with 100 g of distilled water, and then covered with aluminum foils to prevent potential evaporation. The specimens were stored in distilled water at room temperature, taken out at predetermined intervals, dried using a paper towel, and then promptly weighed. Water absorption was determined according to the following formula:

$$WA(\%)=\frac{M_{f}-M_{oi}}{M_{oi}}\times 100$$

where

WA = Water absorption (%),
M_f_ = Sample weight after water absorption (g),
M_oi_ = Oven dry weight after impregnation (g).

## 3. Results and Discussion 

### 3.1. FT-IR of HNTs and Prepared Resins

**Figure 2:**
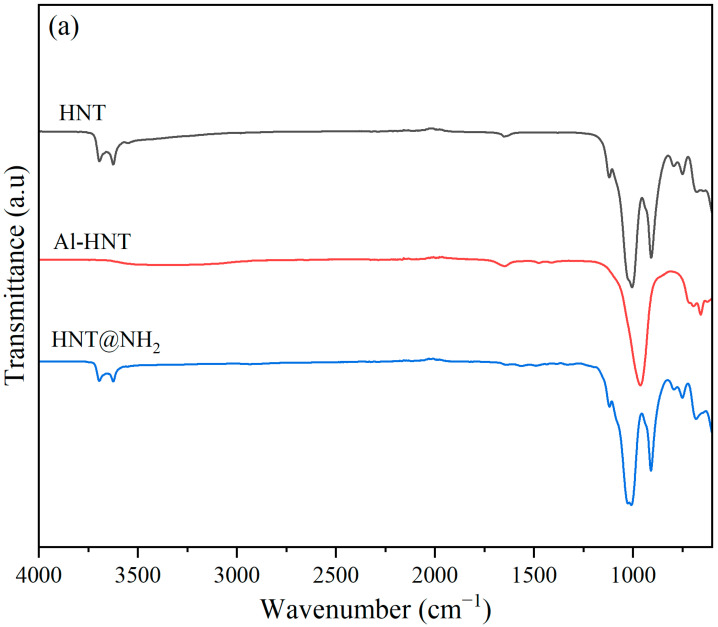

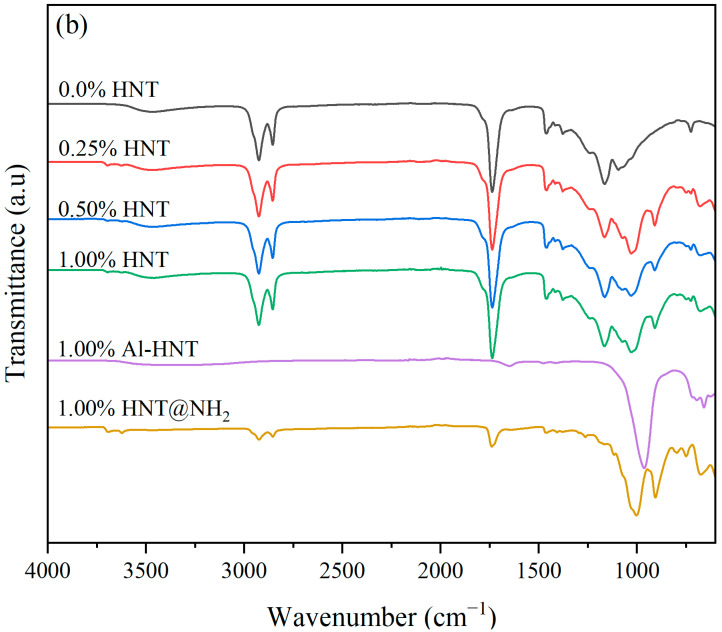
FT-IR spectra of HNT, Al-HNT, and HNT@NH_2_ (**a**) and prepared resins containing different concentrations and types of HNT (**b**).

The FT-IR of HNTs and HNT@NH_2_ are shown in Figure 2a. The characteristic Al-OH absorption bands for the HNTs and HNT@NH_2_ spectra are located at 3622 and 3696 cm^−1^, respectively. This suggests that Hal’s fundamental structure remained unchanged during the silane alteration process [35,36]. Absorption bands that appeared at 1000 cm^−1^ are indicative of O-Si-O stretching vibrations. Peaks at 684, 753, and 910 cm^−1^ correspond to perpendicular Si-O stretching, symmetric stretching of Si-O, and O-H deformation of the inner hydroxyl group, respectively [19]. The absorption band around 1646 cm^−1^ is due to the hydroxyl group (-OH) of the absorbed water [37]. As can be seen, this peak is very weak for HNT@NH_2_, implying less water absorption after modification by APTES. Three weak absorption bands at 2933, 1562, and 1491 cm^−1^ in the spectrum of HNT@NH_2_, are due to the symmetric stretching of C-H_2_, deformation (scissoring) of N-H2, and deformation (scissoring) of C-H_2_, respectively [35,37]. The existence of these peaks attests to the effective grafting of APTES onto the HNTs. In the HNT@NH2 spectrum, the lower intensity of Al-OH bands at 3622 and 3696 cm^−1^ results from the reaction of surface hydroxyl groups with APTES. After treating HNTs with NaOH, the peaks at 3622 cm^−1^, 3696 cm^−1^, and 910 cm^−1^ fully disappear, implying the removal of alumina layers [38,39].

The decrease in the intensity of other peaks related to Si-O bonds reveals that the strong alkali treatment not only removes the aluminum layer but also affects these bonds. As another interesting result, the peak at 1646 cm^−1^, which is related to the hydroxyl group (-OH) of the adsorbed water, shows a slight increase in intensity due to the increase in the amount of absorbed water. The same results were reported before [40]. This might be because the alkali treatment of HNT increases the negative surface charge [23], which increases the attraction of water to the surface of HNT.

Fourier transform infrared spectroscopy (FT-IR) is used to investigate the IR absorption pattern of the prepared resins (Figure 2b). The distinctive functional groups present in the ESOR samples are identified in the spectra. The aliphatic alkanes, (C-H_2_) and C-H_3_, are represented by the peaks found at 2925 cm^−1^ and 2850 cm^−1^, respectively. Furthermore, the distinctive peak of the ether bond is found at 1052 cm^−1^ [41]. The unreacted epoxide groups of epoxidized soybean oil are responsible for the extremely weak peak at 823 cm^−1^. The peak at 725 cm^−1^ is due to the bending vibration of C-H bonds. The peaks at 1098 cm^−1^ and 1166 cm^−1^ are caused by the C-O stretching of aliphatic ethers. A stretching band at 1462 cm^−1^ is attributed to the C-C asymmetric stretching vibration. The sharp peak at 1738 cm^−1^ is due to the C=O bonds of the ester groups of soybean oil [42]. The peak at 1377 cm^−1^ is due to the O-H in-plane bending [12,31]. As can be seen, for films containing HNTs, new peaks at 684, 753, and 910 cm^−1^ appear, which correspond to the perpendicular Si-O stretching, symmetric stretching of Si-O, and O-H deformation of the inner hydroxyl group, respectively [19]. However, the film containing Al-HNT lacks the 910 cm^−1^ absorption band due to the treatment with alkali. The peaks of aliphatic ether C-O stretching (1098 cm^−1^ and 1166 cm^−1^) nearly vanished for films containing HNT@NH_2_ and Al-HNT, indicating a high level of interaction between these nanoparticles and the polymer matrix. However, they remained unchanged when the resins contained unmodified HNTs. A similar rationale can be applied to explain the disappearance of the C=O absorption band at 1738 cm^−1^. 

### 3.2. Transparency and Light Transmission

**Figure 3:**
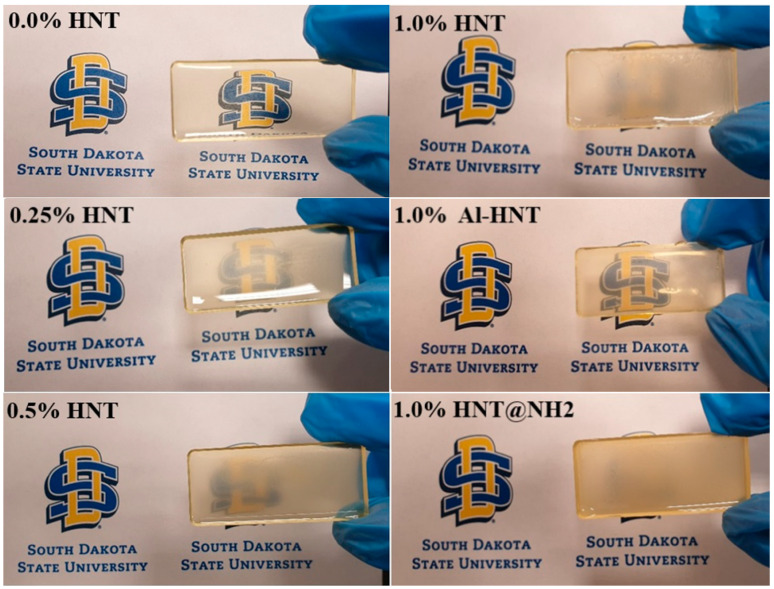
Effect of different concentrations and modification of HNTs on transparency of thermoset resin.

This research examined how the transparency of the thermoset resin blends is affected by HNTs. As shown in Figure 3, different concentrations of HNTs are added to the thermoset resin matrix, ranging from 0.25 wt.% to 1.0 wt.%. It was found that as the concentration of HNTs increases, the transparency of the blend decreases, and at a concentration of 1.0 wt.%, the resin becomes entirely opaque. This reduction in transparency can be attributed to the HNTs aggregation, which reduces resin transparency by diffracting incident light [43]. HNTs are prone to aggregation due to their limited interfacial compatibility with the hydrophobic epoxy resin matrix, high aspect ratio, and hydroxyl surface groups. Two alternative modification techniques are used to address the poor dispersion and aggregation of HNTs in the thermoset resin matrix: sodium hydroxide treatment and (3-Aminopropyl) triethoxysilane (APTES) functionalization. HNTs’ surface charge is known to increase after treatment with sodium hydroxide, which enhances their ability to disperse in the resin. As shown in Figure 2, despite the unmodified HNT, at 1.0 wt.%, the alkali-modified HNT forms clear blends, suggesting enhanced HNT dispersion and decreased aggregation in the thermoset resin matrix. This improvement is explained by the increased surface charge of HNTs after treatment with sodium hydroxide, which keeps the HNTs apart and enhances their uniform dispersion in the resin matrix [23].

On the other hand, compared to the unmodified HNTs, APTES functionalization does not result in an improvement in transparency. This surprising finding raises the possibility that APTES functionalization may not completely prevent the aggregation of HNTs in the resin matrix or could introduce new elements that impact the transparency of the blends. In other words, introducing APTES worsens the aggregation of HNTs in ESOR, resulting in a loss of transparency. The authors believe that in the ESOR environment, compatibility between APTES-modified HNTs is more about their compatibility with ESOR. Hence, they tend to aggregate to minimize their interaction with ESOR. These results underline how crucial it is to choose the HNTs’ modification technique carefully and optimize it to achieve the desired results in thermoset resin blends. For instance, the modification of HNTs with APTES can improve their dispersion in polyethylene terephthalate (PET), as shown in this reference [44]. They used an X-ray diffraction analysis to indicate the improved dispersion of HNTs in PET results from improved compatibility between HNTs and PET. To further improve compatibility between nanoparticles like HNTs, APTES modification can be utilized to graft the desired polymer onto the nanoparticle’s surface, where the free-NH_2_ can act as an initiator for polymer grafting [45]. 

**Figure 4:**
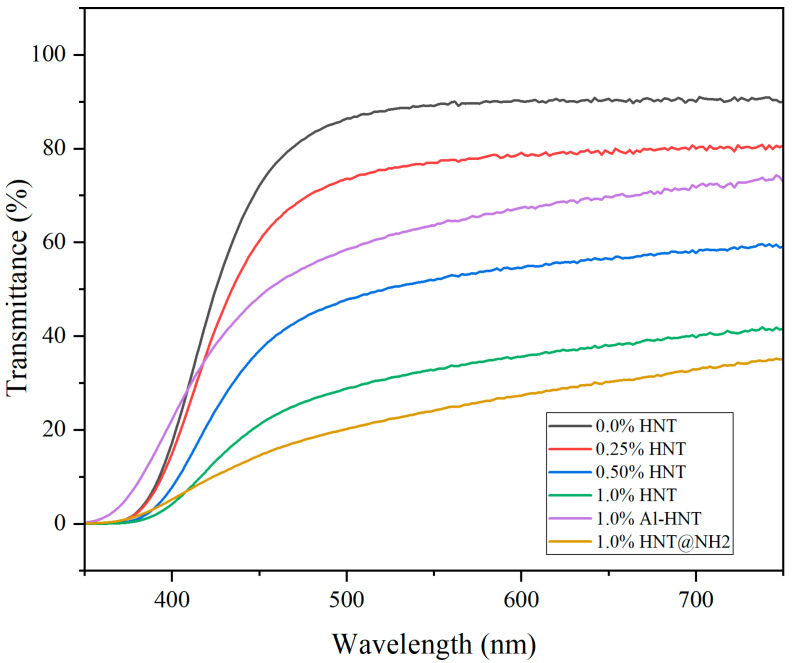
Light transmittance of prepared resins with different concentrations and types of HNTs.

The same pattern is observed in the light transmittance of the prepared ESOR blends (Figure 4). As shown in Figure 4, the resin containing HNT@NH_2_ displays the minimum visible light transmittance. However, the ESOR sample containing 1 wt.% of Al-HNT displays higher transmittance than ESOR with 0.5 wt.% of non-modified HNTs. This suggests that Al-HNT will disperse more effectively even at twice the concentration of the unmodified HNTs.

**Figure 5:**
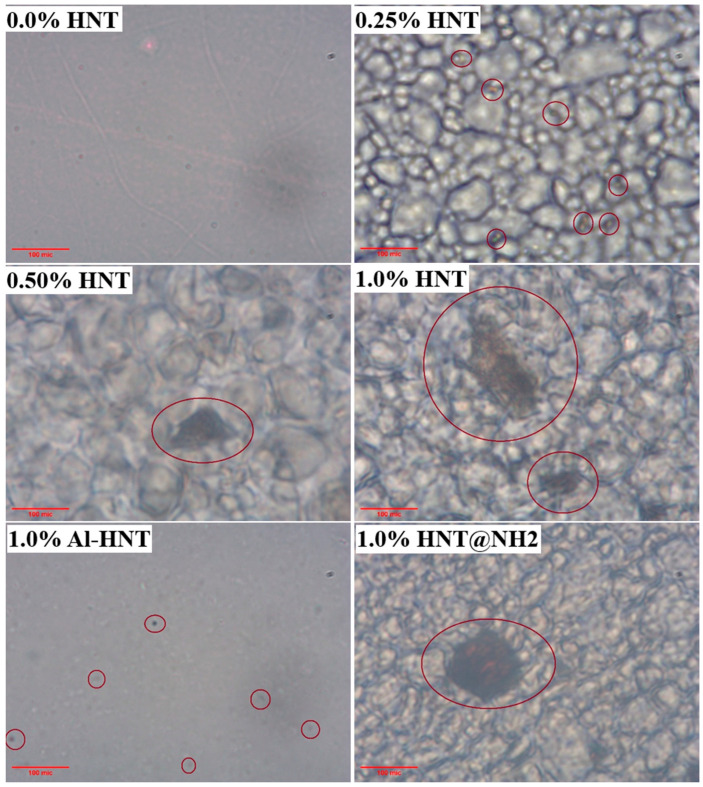
Micrograph images of prepared resins with different concentrations and types of HNTs. Red circles point to the aggregated HNTs.

To further investigate the added HNTs at the micron level, the ESOR samples are studied using an optical microscope, and the obtained images are shown in Figure 5. As can be seen, the size of the aggregated HNTs increases with their concentration in the ESOR. It is interesting to note that when compared to the unmodified HNTs, the aggregated particles of Al-HNT are far smaller. Because of their small size, it is challenging to find them with an optical microscope. For HNT@NH_2_ particles, the observed sizes are almost the same as the unmodified ones, though the shape of the particles is spherical. The authors believe the change in the shape of the aggregated particles of HNT@NH_2_ to spherical results from the incompatibility of the alkyl part of APTES with their surroundings and their tendency to minimize an undesirable interaction with the ESOR matrix.

### 3.3. Mechanical Strength

**Figure 6:**
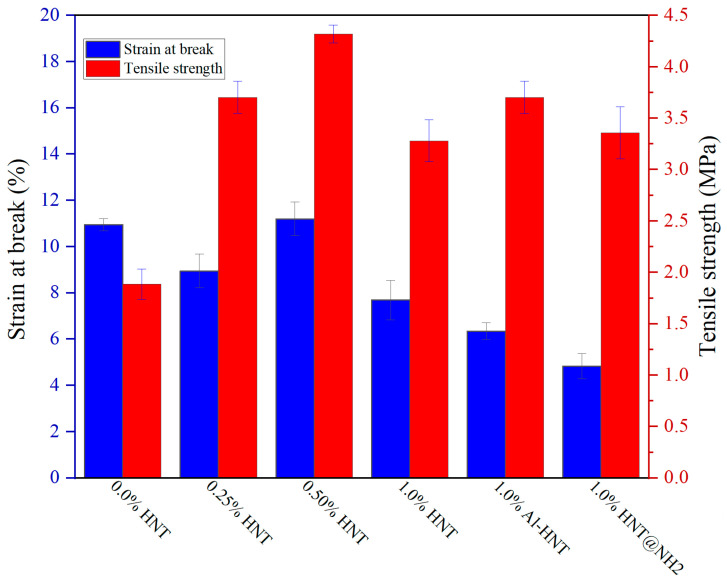
Mechanical strength of prepared resins containing different amounts of HNTs.

The effect of HNTs’ concentration and type on the mechanical strength of the prepared ESOR is shown in Figure 6. Based on the experimental results, it is shown that the amount of HNTs significantly affects the mechanical strength of the ESOR. Tensile strength measurements indicate that the mechanical strength of the ESOR increases with the addition of HNTs up to a concentration of 0.5 wt.%. However, the mechanical strength of the ESOR decreases at a concentration of 1.0 wt.%. The reinforcement offered by HNTs, which have a high aspect ratio and might form a network structure inside the resin matrix, is responsible for the increase in mechanical strength. The more stress transfer spots available in the ESOR, the better the mechanical strength. However, at high concentrations, HNTs may aggregate and create stress concentration points, resulting in a reduction in mechanical strength. The extent of HNTs’ aggregation depends on several variables, including the concentration of the filler, the method of dispersion, and the filler–matrix interaction. To prevent aggregation and ensure optimal mechanical performance, it is crucial to carefully regulate the concentration and dispersion of the HNTs within the matrix [19,46]. As shown, the ESOR containing 1.0 wt.% of Al-HNT displays the maximum mechanical strength and displacement, highlighting the importance of the proper dispersion of nanoparticles in the resin film to take full advantage of its mechanical properties. The same concentration of HNT@NH2 (1.0 wt.%) results in a small improvement in tensile strength, while it reduces the elongation (displacement) compared to the pure ESOR. It seems the amino groups of APTES, at the surface layer of aggregated HNT@NH2 particles, strongly interact with the functional group of the ESOR, as shown in FT-IR results, restricting their movement. This restricted movement of the polymeric chain of the resin leads to lower elongation.

### 3.4. Thermal Gravimetric Analysis 

**Figure 7:**
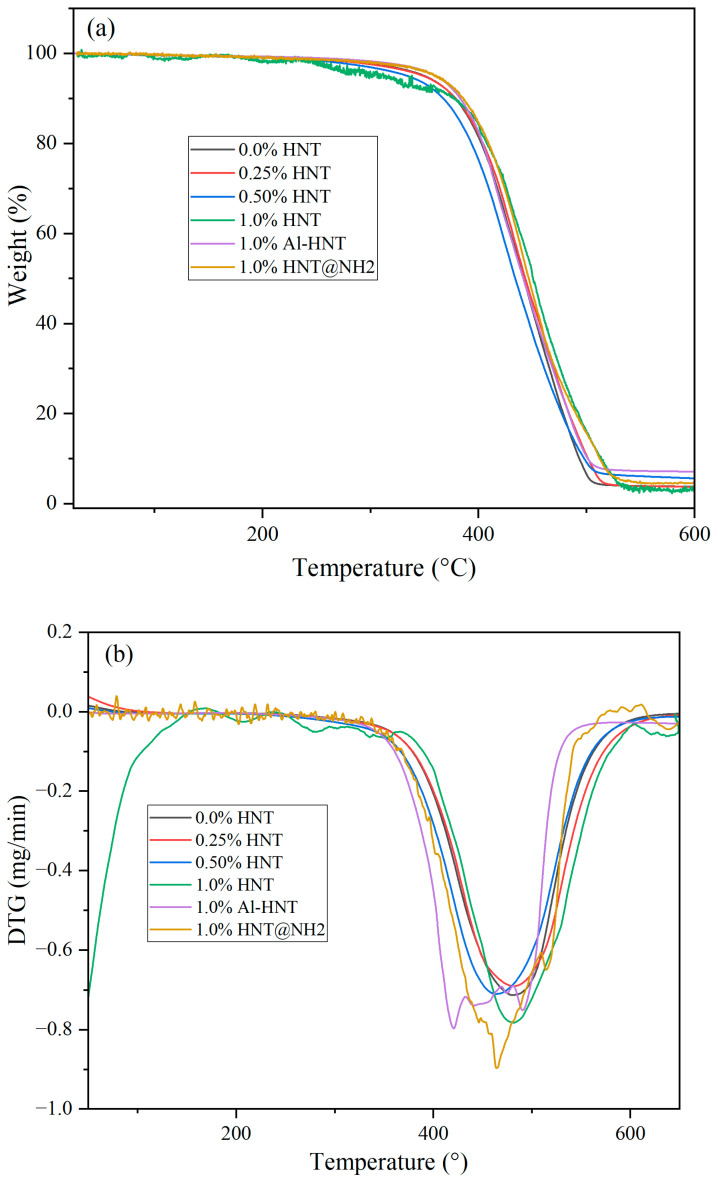
TGA (**a**) and DTG (**b**) thermograms of prepared resins.

The influence of the concentration and type of halloysite nanotubes (HNTs) on the thermal characteristics of epoxidized soybean oil resin (ESOR) is examined using a thermogravimetric analysis (TGA) and its derivative, DTG, as shown in Figure 7. The results indicate that both the pure resin and the resin containing 0.25 wt.% HNT exhibit the same onset temperature for thermal degradation. In contrast, the resin with 0.50 wt.% HNT shows a slightly lower onset temperature for thermal degradation. However, when the concentration of HNT is increased to 1.0 wt.%, regardless of the type of HNT, the resin demonstrates the highest onset temperature for thermal degradation. This suggests a slight improvement in the thermal stability of the resin at higher HNT concentrations.

The enhancement in thermal stability can be attributed to a combination of factors. First, the high aspect ratio of HNTs acts as a barrier to the diffusion of oxygen and other gases, reducing the rate of thermal degradation. Second, the unique morphology of HNTs, with their porous interior and closed extremities, can capture volatile degradation products during heating and prevent their release. This can decrease the rate of mass loss and enhance thermal stability [46]. Additionally, the interaction between HNTs and the ESOR matrix can contribute to the enhancement of thermal stability. The hydroxyl groups on the surface of the HNTs can form hydrogen bonds with the epoxy functional groups, resulting in a stronger interfacial interaction and improved thermal stability [19,46]. As shown in Figure 6, the modification of HNTs does not affect the thermal stability of the final ESOR compared to the unmodified ones. 

### 3.5. Water Absorption Capacity 

Epoxy resins are extensively employed in various sectors, including aerospace, automotive, construction, electronics, and coatings. A crucial characteristic of epoxy resins is their propensity to absorb water, which leads to a reduction in their mechanical strength and dimensional stability and can significantly impact their performance and durability in various applications. The water absorption capacity refers to the ability of resin to absorb water molecules from the surrounding environment, resulting in swelling, softening, and degradation of the resin matrix. This reduces the mechanical strength, stiffness, and adhesion of epoxy resins, thus diminishing their performance and service life. Epoxy resins may be treated with appropriate surface treatments or coatings, hydrophobic additives, surface-modified nanoparticles like halloysite, optimized curing processes, and other techniques to minimize water absorption [47,48,49].

**Figure 8:**
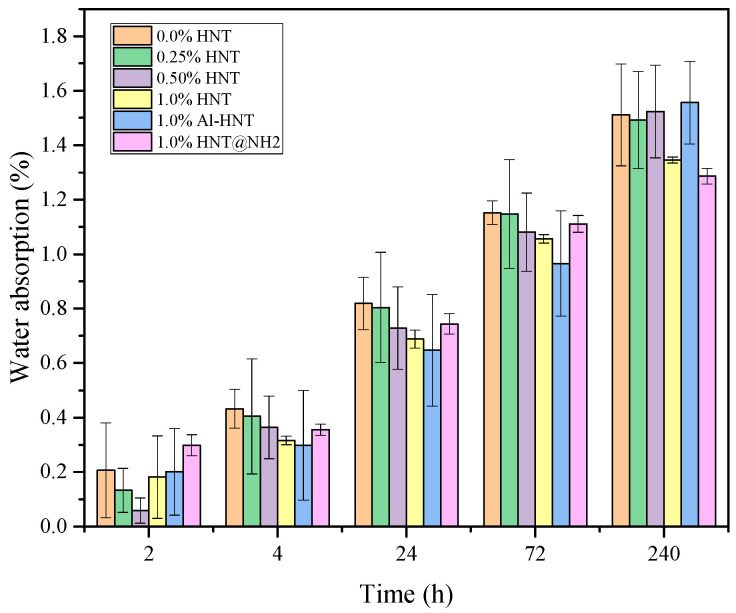
The change in water absorption of prepared resins with time.

The water absorption capacity of the prepared ESOR is presented in Figure 8. The water absorption values of the resin samples with different amounts and types of halloysite nanoparticles (HNTs, Al-HNT, and HNTs@NH_2_) are within an acceptable range from 1.28% to 1.55%. When no HNTs are added (0.0 wt.% HNT), the epoxy resin blend exhibits an average water absorption ranging from 0.206% to 1.511% for 2–270 h. As the amount of HNTs increases to 1.0 wt.%, the average water absorption generally decreases. 

The dispersed HNTs fill the voids within the ESOR matrix and act as a barrier, prohibiting the diffusion of water molecules into the polymer matrix. In the case of Al-HNTs, the average water absorption is somewhat greater than that of pure epoxy resin. This may be because the presence of strongly negatively charged HNTs increases the water hydrophilicity of the resin, which in turn causes the water absorption to be slightly higher. The ESOR containing 1.0 wt.% of HNT@NH_2_ exhibits the lowest average water absorption at the time of 240 h. This is due to the silane modification of HNTs, which reduces their hydrophilicity substantially, as shown in the FT-IR results. These results emphasize the significance of incorporating modified nanoparticles, such as HNT@NH_2_, into the ESOR to obtain a lower water absorption property, which is essential for assuring the long-term performance and durability of epoxy-based materials in a variety of applications.

## 4. Conclusions

The effect of the halloysite concentration on the mechanical strength of soybean oil-based thermoset resins was investigated to explore their future application in the medical and healthcare sectors. It was found that the addition of halloysite nanotubes (HNTs) at 0.25 and 0.50 wt.% led to an increase in mechanical strength, while the addition of HNTs at 1.0 wt.% decreased the mechanical strength. NaOH and APTES treatments were used to modify the surface of the HNTs. The results showed that the addition of NaOH-treated HNTs improved the transparency and mechanical strength of the blend films, indicating better dispersion of the filler in the resin matrix. However, the addition of APTES-treated HNTs worsened the mechanical strength while improving the water resistance of the prepared ESOR. The incorporation of halloysite nanotubes enhanced the performance of the soybean oil-based epoxy resin, making it suitable for advanced medical packaging applications. This research opens new possibilities for the development of sustainable, high-performance, and eco-friendly materials that can ensure the safety and integrity of medical devices and products.

## Figures and Tables

**Table 1:** ESOR composition with HNTs or functionalized HNTs.

Sample	Filler (g)	Matrix	
-	-	ESO (g)	Acrylic Acid (g)
0.0% HNT	0.0 HNTs	50	50
0.25% HNT	0.125 HNTs	49.875	50
0.50% HNT	0.250 HNTs	49.75	50
1.0% HNT	0.50 NTs	49.5	50
1.0% Al-HNT	0.5 Al-HNT	49.5	50
1.0% HNT@NH_2_	0.5 HNT@NH2	49.5	50

## Data Availability

Data are contained within the article..

## Abbreviations

Al-HNT	Alkali-modified halloysite nanotubes
APTES	(3-Aminopropyl) triethoxysilane
DES	Deep eutectic solvent
ESO	Epoxidized soybean oil
ESOR	Epoxidized soybean oil resin
FT-IR	Fourier transform infrared
HNTs	Halloysite nanotubes
HNT@NH2	(3-Aminopropyl) triethoxysilane-modified halloysite nanotubes
PLA	Polylactic acid
PVC	Polyvinyl chloride
SO	Soybean oil
WA	Water absorption
